# Diffusion through Pig Gastric Mucin: Effect of Relative Humidity

**DOI:** 10.1371/journal.pone.0157596

**Published:** 2016-06-23

**Authors:** Anna Runnsjö, Aleksandra P. Dabkowska, Emma Sparr, Vitaly Kocherbitov, Thomas Arnebrant, Johan Engblom

**Affiliations:** 1 Biomedical Science, Faculty of Health and Society, Malmö University, Malmö, Sweden; 2 Biofilms—Research Center for Biointerfaces, Malmö University, Malmö, Sweden; 3 Division of Physical Chemistry, Lund University, Lund, Sweden; University of Manchester, UNITED KINGDOM

## Abstract

Mucus covers the epithelium found in all intestinal tracts, where it serves as an important protecting barrier, and pharmaceutical drugs administrated by the oral, rectal, vaginal, ocular, or nasal route need to penetrate the mucus in order to reach their targets. Furthermore, the diffusion in mucus as well as the viscosity of mucus in the eyes, nose and throat can change depending on the relative humidity of the surrounding air. In this study we have investigated how diffusion through gels of mucin, the main protein in mucus, is affected by changes in ambient relative humidity (i.e. water activity). Already a small decrease in water activity was found to give rise to a significant decrease in penetration rate through the mucin gel of the antibacterial drug metronidazole. We also show that a decrease in water activity leads to decreased diffusion rate in the mucin gel for the fluorophore fluorescein. This study shows that it is possible to alter transport rates of molecules through mucus by changing the water activity in the gel. It furthermore illustrates the importance of considering effects of the water activity in the mucosa during development of potential pharmaceuticals.

## Introduction

Mucus gels exist on the luminal surface of the gastrointestinal, urogenital and respiratory tract as well as on the cornea of the eye. The gel acts as a lubricant, serves as a protective barrier against harmful agents and microbes and ensures that hydration of underlying tissue is maintained [1–4]. Mucus consists mainly of water (95%), lipids, proteins, DNA and salt. Mucins, the major protein family in mucus, is responsible for the gel forming properties of mucus [1,2,5]. These large glycoproteins display a vast polydispersity in both mass and size [1] and can assemble into long chains [6–8], bridged together by disulfide bonds. Entanglement of these long mucin chains is considered to be the primary mechanism for gel formation [1,6]. Therefore, the mucin concentration is one major factor which determines the gel properties of mucus [9–13]. Investigations of mucin physiochemical behavior have shown that the hydration level also affects the phase behavior of mucin to a large degree. Rheology measurements have shown that the viscoelastic properties of mucin are largely dependent on the mucin concentration where mucin solutions from gels between 4 to 5 wt% [9,10,13]. However factors such as pH and salt concentration also affects the biophysical properties of mucin in water and hence the diffusion rates of substances therein [9,11,12,14]. Mucin gels furthermore undergo a glass transition (Tg) which for mucin is independent of the hydration level in gels with less than 67 wt% mucin (Tg -15°C), but increases in temperature at higher mucin concentrations. This implies that dilute mucin gels will dehydrate and pass through the glass transition when subjected to a relative humidity below 60–70%RH at ambient temperature (corresponding to a mucin concentration of 80–85 wt%) [15,16], going from an elastic to a glassy state, which for many polymers has a high impact on diffusion in the gel [17].

As mucus is the first barrier which nutrients and enteric drugs must pass through during uptake into the body [1,3,18], it is highly relevant to investigate diffusional transport through mucus films during e.g. drug development. Diffusion of various molecules and particles through highly diluted mucin gels and mucus have indeed been thoroughly investigated [3,5,18–26]. It is generally found that molecules and particles diffuse slower in mucus than in water. The diffusion rate is depend on size, hydrophobicity and surface charge of the molecules/particles. [18,19,21,23–25]. Furthermore diffusion in mucus is limited both due to the formed mucus mesh and the interaction between the particles/molecules and the mucus components [3,19]. The mucus mesh is reported to be heterogeneous with pore sizes from 20 to 200 nm and up to micrometer sized, depending on the mucus concentration, pH and the origin of the gel [2,3,23,24,27–29].

Most previous studies have investigated diffusion in mucus containing relatively large amounts of water, 90–100% [9–13,13,26]. However, mucus films covering the eyes, nose and throat become dry when exposed to environments with low relative humidity [30]. Additionally, application of a mucoadhesive formulations might reduce the water content in the mucus film [31]. The permeability of mucus changes with concentration [9–14], which makes it relevant to investigate drug transport through mucus exposed to a drier environment causing dehydration and increased mucin concentration.

Previously it has been shown that biological membranes such as skin and buccal mucosa respond to changes in ambient water activity [32,33]. A change in water activity can indeed act as a switch to regulate the permeability of these membranes [32], resulting in high permeability at high water activities and low permeability at low water activities. As the mucosal barrier consists of both a mucus film and a layer of epithelial cells, the permeability is regulated by the barrier properties of both the mucus and the underlying tissue. To further elucidate the barrier mechanism behind the mucus layer we here investigate the permeability through mucin gels.

The aim was to evaluate how the water activity affects diffusion of small molecules in highly concentrated mucin gels. Decreased water activity also leads to decreased water content in the gel [15,16]. Furthermore when at equilibrium, the water activity (a_w_) in the mucin gel is related to the relative humidity (RH) of the surroundings according to a_w_ = RH/100 and to the osmotic pressure (Π_osm_) according to Π_osm_ = -RT/V_w_*ln(RH/100) where R is ideal gas constant, T is the temperature and V_w_ is the molar volume of water [34]. We used three complementary techniques to investigate how the water activity affects molecular diffusion in pig gastric mucin gels. We report how the transport of the anti-bacterial drug metronidazole, which is commonly used for treatment of bacterial infections in mucus containing tissue, is affected by reduction in water activity in the mucin film from 1 to 0.85. Utilizing fluorescence recovery after photobleaching (FRAP) and fluorescence correlation spectroscopy (FCS), we also determined the diffusion coefficient for a small hydrophilic molecule, fluorescein, in mucin gels with water activities down to 0.12.

## Materials and Methods

### Materials

Metronidazole was obtained from Mediolast (Italy) and sodium fluorescein was purchased from BDH Chemicals (UK). Pig gastric mucin type III was obtained from Sigma Aldrich (Sweden) and used as received. Polyethylene glycol 4000 Da (PEG) (ultra grade, Fluka), used to regulate the water activity in metronidazole penetration experiments, was also sourced from Sigma Aldrich (Sweden). Atto 488, used for calibration of the FCS experiment, was purchased from Sigma Aldrich (Germany). Ultrapure water with a resistivity of 18.4 MΩ cm was used in all experiments.

### Preparation of mucin and PEG gels for penetration studies

Mucin gels were prepared by dispersing mucin in water to a concentration of 50 wt%. After equilibration for at least 24 h the gels were placed between a silicone membrane (Specially Manufacturing Inc., USA) and a Millipore GV 0.22 filter paper (Merck Millipore, USA) using a 1.74 mm thick FEP coated O-ring (Lamisa Teknik AB, Sweden) as spacer, all thoroughly cleaned with ultrapure water.

To create PEG gels with water activities of 0.996, 0.981, 0.930 or 0.867 at 32°C PEG was dispersed in ultrapurified water to a concentration of (5, 20, 45 or 60 wt% [32]). The gels were inserted between silicone and Millipore GV 0.22 membranes, separated by a 1.74 mm thick O-ring.

The mucin and PEG gels, deposited between the membranes, were inserted in the flow through cells with the silicone membranes exposed to the receptor solution and the Millipore membrane exposed to the donor chamber. The water activity of the gels was regulated by addition of aqueous PEG solutions with water activities of 0.996, 0.981, 0.930 or 0.867 to the donor chambers. The gels were then left to equilibrate in this setup for at least 2 h before the donor solutions were exchanged to PEG solutions containing metronidazole and the penetration experiment was initiated (t = 0 h). The concentration of metronidazole in the donor chamber was set to a saturation level of 0.9 for all used PEG concentrations.

### Flow through diffusion cells

Penetration experiments were performed with flow-through cells [35], a system designed by Clowes et al. [36]. Gels with a diffusion area of 0.64 cm^2^ (9 mm ID) were used. To avoid air bubbles under the gel the receptor phase was degassed at low pressure for minimum 30 min before it was pumped (Ismatec IPC-16, IDEX Health & Science, Germany) through the system at a flow rate of 1.5 mL/h. The receptor solution which comprised of ultrapure water was stirred with PTFE coated magnetic bars (7×2 mm). The temperature was controlled at 32±0.3°C (Techne TE-10A Thermoregulator, Bibby Scientific, UK) by circulating water through a heat block surrounding the flow through cells. Fractions of receptor solution were collected (Gilson FC 204 Fraction collector, Gilson Inc., USA) at appropriate time intervals throughout the experiment. The cells were occluded with parafilm (Pechiney Plastic Packaging Company, USA) to avoid evaporation during the experiment.

### Metronidazole analysis

The amount of metronidazole which passed the membranes in the penetration studies was analyzed spectrophotometrically using Varian Cary 50 Bio UV- visible spectrophotometer (Agilent Technologies). Detection wavelength was 320 nm and calibration curves of standard solutions (0.3–20.0 μg/mL metronidazole) were used to calculate the concentration in each assembled receptor sample.

### Preparation of concentrated mucin films for FCS and FRAP experiments

Mucin films with water activities between 0.12 and 0.97 were made by equilibrating mucin solution in chambers with fixed relative humidity. This gave rise to gels containing 50 wt% to 97 wt% mucin as previously determined by water sorption isotherms [15,16], reported in Table 1 and S1 Table. The samples were prepared as follows. Mucin was dispersed in water containing 0.1 wt% sodium azide, to prevent microbial growth, and 100 μM or 10 nM sodium fluorescein to a concentration of 10 wt%. The mucin was then placed on microscope slides thoroughly cleaned with ethanol and ultrapure water. For FRAP measurements Chance Propper Cover glass No. 1 (Fisher Scientific, Sweden) were used and for FCS measurements #1.5 cover glasses (Menzel-Glaser, Menzel GmbH & Co., Germany) were used. A ring of modeling clay (FIMO soft, STAETLER Mars GmbH & Co. KG, Germany) was used to prevent the mucin solution from float out. The mucin gels were then equilibrated at 25°C for at least one week in desiccators containing saturated salt solutions (LiCl, MgCl_2_, NaBr, SrCl_2_, NaCl, KBr, KCl, KNO_3_ and K_2_SO_4_) to adjust the humidity and hence the water activity in the mucin gels. These salt solutions give a relative humidity of 11, 33, 57, 71, 75, 81, 84, 93 and 98% at 25°C [37,38]. Using water sorption isotherms previously reported [15,16] the mucin concentration in the film was calculated, S1 Table. To maintain the water concentration in the mucin films during FRAP and FCS measurements the films were sealed using a second microscope slide. The water activity in the mucin gels at 32°C was calculated using water sorption isotherms for mucin at 25°C and 40°C [16]. The mucin samples were thereby found to have a water activity of 0.12, 0.35, 0.66, 0.73, 0.77, 0.81, 0.85, 0.94 and 0.97at 32°C.

**Table 1:** Properties of the donor formulations and the gels comprised between the membranes, followed by resulting steady state flux (J_ss_).

Donor formulation			Polymer between membranes	Flux
**wt% PEG**	a_w_* at 32°C	metronidazole solubility* (mg·ml^-1^)		J_ss_ (μg·cm^-2^·h^-1^)
**5**	0.996	11.2 ± 0.1	n.a.	2.6 ± 0.1
**20**	0.981	12.1 ± 0.1	n.a.	2.6 ± 0.1
**45**	0.930	15.2 ± 0.5	n.a.	2.5 ± 0.3
**60**	0.867	18.7 ± 1.0	n.a.	2.3 ± 0.5
**5**	0.996	11.2 ± 0.1	5 wt% PEG	2.2 ± 0.2
**20**	0.981	12.1 ± 0.1	20 wt% PEG	2.2 ± 0.2
**45**	0.930	15.2 ± 0.5	45 wt% PEG	1.6 ± 0.3
**60**	0.867	18.7 ± 1.0	60 wt% PEG	1.9 ± 0.3
**5**	0.996	11.2 ± 0.1	<50 wt% mucin **	2.4 ± 0.3
**20**	0.981	12.1 ± 0.1	<50 wt% mucin **	2.2 ± 0.5
**45**	0.930	15.2 ± 0.5	63 wt% mucin **	1.2 ± 0.5
**60**	0.867	18.7 ± 1.0	68 wt% mucin **	0.4 ± 0.2

* Addapted from Björklund et al. [32]

** Approximate mucin content based on water sorption isotherms by Znamenskaya et al [15,16].

### Fluorescence microscopy

Fluorescence recovery after photo bleaching (FRAP) and fluorescence correlation spectroscopy (FCS) measurements were carried out on a SP5 scanning confocal microscope (Leica, Germany) equipped with Pico Harp 800 FCS setup (PicoQuant, Germany). For FRAP experiments a 63x glycerol immersion objective with numerical aperture (n.a) of 1.3 was used while FCS measurements were carried out using 63x water immersion objective (n.a. of 1.2) with the pinhole diameter set to airy unit 1. The temperature was regulated to 32°C by an air cooler (Fyrka Kältechik GmbH, Germany). Sodium fluorescein was excited using a 488 nm Argon laser and emission was captured between 499–533 nm. For FCS measurements the laser intensity was set to nominal 60% (at 30% tube power).

### FCS analysis

FCS measures fluctuations in the measured fluorescence intensity, F(t), caused by diffusion of fluorescein molecules in and out of the confocal volume. The autocorrelation function G(τ) compares the fluorescence intensity, F, at time t with the intensity after a lag time (t + τ)[39].

$$G(\tau )=\frac{\left\langle F(t)\rangle \langle F(t+\tau )\right\rangle }{{\left\langle F(t)\right\rangle }^{2}}$$ (1)

The symbols <…> imply averaging over a long time period (e.g. data points in the time series). The autocorrelation function can be simplified to

$$G(\tau )=1+\frac{A}{\left(1+\frac{\tau }{\tau_{D}}\right)}\sqrt{1+\kappa^{2}\frac{\tau }{\tau_{D}}}$$ (2)

where A is the amplitude contributed by the fluorophores, κ is a structural parameter relating the axial and radial radius of the confocal volume and τ_D_ is the diffusion time which is the time the molecule spend in the confocal volume. The diffusion time is further related to the diffusion coefficient according to

$$D=\frac{\omega^{2}}{4\tau_{D}}$$ (3)

where ω is the radius of the confocal volume [39].

The autocorrelation curves were analyzed using the SymPhoTime software (PicoQuant, Germany) by fitting to pure diffusion model and fixing the focal volume. The focal volume was determined by measuring a sample with a known diffusion coefficient (Atto488 or fluorescein), where the temperature-adjusted diffusion coefficient for Atto488 is 478 μm^2^/s at 32°C and 510 μm^2^/s for fluorescein [40,41]. The FCS measurements were performed in three locations, each at least 20 μm from the microscope slide surface.

### FRAP analysis

During FRAP measurements a circle with a diameter of 10 μm was bleached in the mucin gels. The focal plane was set to 15–30 μm from the glass surface in order to measure in the bulk of the mucin gels, found to be approximately 30–80 μm thick. With these settings most of the film in the z direction was bleached (see S1 Fig). Hence, we can assume the diffusion to be 2-dimensional. The recovery of the intensity, I(t), in the bleached spot was modeled to a single exponential function according to [42]

$$I(t)/I_{0}=1-{Ae}^{-\beta t}$$ (4)

where I_0_ is the fluorescence intensity before bleaching, A is the fraction of fluorophores which are bleached, β is a constant which determines the recovery rate and t is the time in seconds. The fraction of bleached molecules can be calculated from intensity of the first measurement after bleaching (I_1_) and the intensity before bleaching (I_0_) according to

$$A=(I_{0}-I_{1})/I_{0}$$ (5)

Rearranging Eq 4 gives

$$\ln\left(I_{0}-I(t)\right)=\ln\left(I_{0}-I_{1}\right)-\beta t$$ (6)

and β can hence easily be found as the slope in the plot of ln(I_0_ –I(t)) against t. Furthermore, to account for differences in total fluorescence during time course of the experiment changes in the baseline were modified by subtracting baseline deviations measured in a reference spot outside the bleached area. Finally, the half time of recovery is obtained as

$$t_{1/2}=\ln\left(2\right)/\beta$$ (7)

and according to Axelrod et al. [43] the diffusion coefficient from a circular bleached spot can be obtained from

$$D=\frac{\omega^{2}}{4t_{1/2}}\gamma$$ (8)

where *ω* is the radius of the bleached spot and γ a constant which is typically 0.88 for circular spots, assuming that no recovery occur during the bleaching. This approximation may give rise to a slight underestimation of the diffusion coefficient for the mucin gels with a water activity of 0.97 where diffusion occur during bleaching. However, the heterogeneity in the mucin gels made it difficult to use other methods like the Hankel transform method [44] for the analysis. All data analysis of the recovery curves were performed in Matlab (MathWorks, USA).

## Results

The overall aim of this study was to evaluate how the water activity affects diffusion through mucin gels. Using flow though diffusion cells we investigated how the penetration rate of the anti-bacterial drug metronidazole changes as a function of the water activity (a_w_) in the mucin gel. In addition, we have also investigated how the diffusion coefficient of fluorescein varies with the water activity in mucin gels using fluorescence recovery after photobleaching (FRAP) and fluorescence correlation spectroscopy (FCS) measurements.

### Penetration of metronidazole

**Fig 1:**
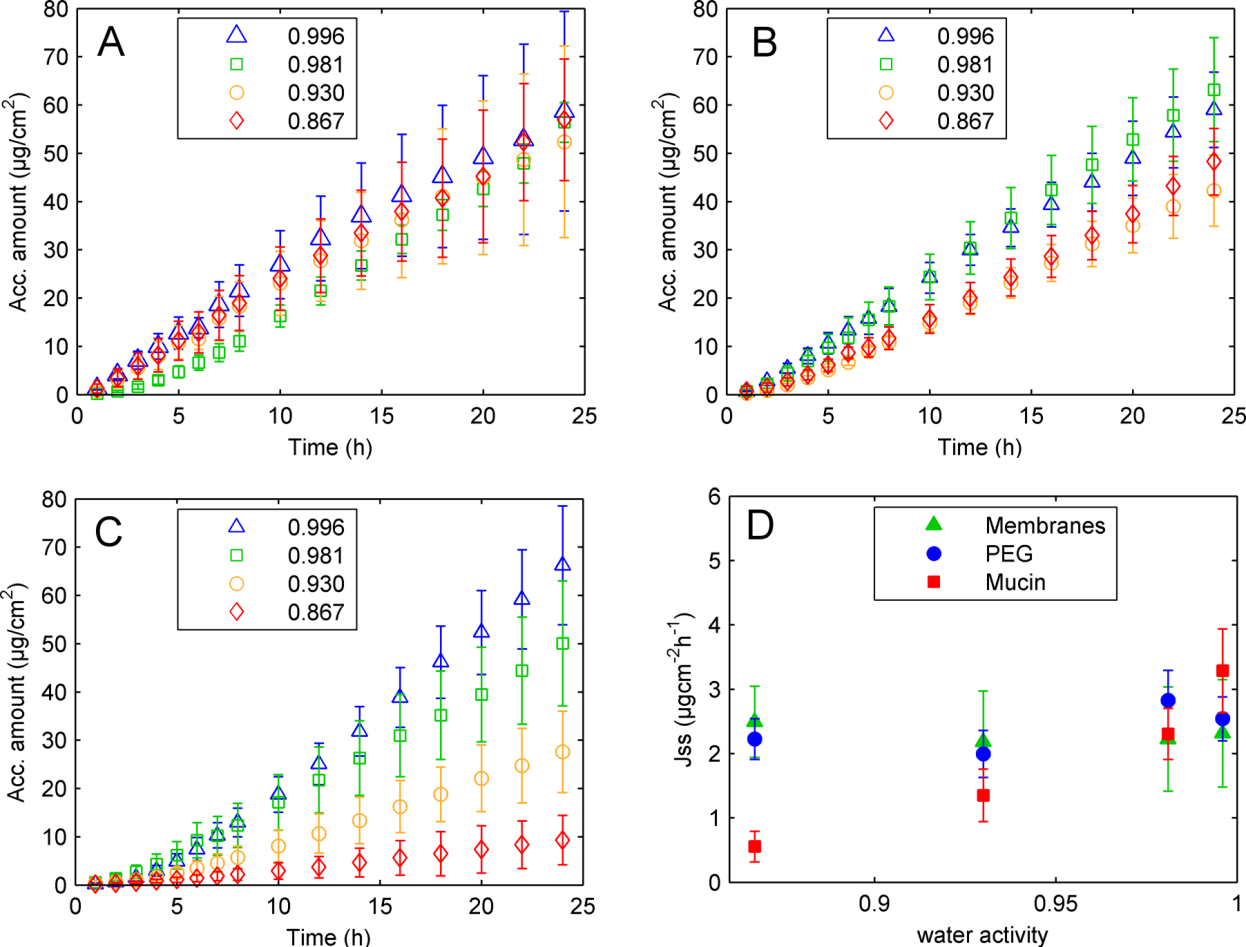
Penetration of metronidazole through polymer gels with different water activities. Accumulated amount of metronidazole penetrated through combined silicone and Millipore GV membranes alone (A) separated by PEG gels (B) or mucin gels (C) is shown vs water activity. Water activity of donor formulations, which also regulated the water activity of the gels enclosed between the membranes, was 0.996 (blue open triangles), 0.981 (green open squares), 0.930 (yellow open circles) and 0.867 (red open diamonds). Steady state flux, calculated over 6–24 h from data provided in Fig 1A to CC, versus water activity for membranes only (green filled triangles), PEG gels (blue filled circles) and mucin gels (red filled squares) are shown in D.

Fig 1 shows how the water activity of the surroundings affects penetration of metronidazole through PEG and mucin gels. For this study a flow through cell setup, previously described [32,35,36], was used. The mucin gel was separated from the donor and receptor compartment with a Millipore GV0.22 filter and a silicone membrane, respectively. The donor compartment was filled with four different concentrations of PEG 4000 (5, 20, 45, or 60 wt%), which gave rise to water activities between 0.996 to 0.867 at 32°C, Table 1 [32], while the receptor compartment was filled with water. Assuming that water penetration through the silicone membrane is small compared to water penetration through the Millipore GV0.22 filter, the water activity in the mucin gels is regulated by the water activity in the donor compartment. The activity of the drug in the donor formulations can be assumed to be constant with time as less than 0.5% of the applied metronidazole penetrated the membranes within the experimental time. Furthermore, the concentration of metronidazole in the different donor solutions was regulated with respect to the saturation level of metronidazole in the PEG solutions. Thereby the activity of metronidazole was the same in all donor solutions, allowing us to compare the transport of metronidazole through mucin gels as a function of the water activity in the gel.

The diffusion rate of metronidazole through mucin gels was found to strongly depend on the water activity in the mucin gel (Fig 1C). The release rate of metronidazole from donor solutions was however the same for all donor solutions investigated. This is implied from the data in Fig 1A where the penetration of metronidazole through the silicone and Millipore GV 0.22 membranes are shown. No difference in penetration rate of metronidazole was observed in this case. Therefore the observed decrease in metronidazole penetration rate through mucin with decreased water activity is due to changes in the mucin gels. We also investigated the penetration rate of metronidazole through PEG gels (Fig 1B) to validate the experimental design and to have a reference to compare our mucin data with. When PEG gels were inserted between the membranes instead of mucin gels no change in metronidazole penetration rate was observed with changes in water activity in the gel. Hence the changes in the penetration rate of metronidazole through polymer gels with decreased water activity is not a generic property all polymers, but will depend on polymer composition.

For metronidazole penetration through mucin gels shows a lag time of about five hours (Fig 1C). Although the gels were incubated with PEG solutions in the donor compartment for 2 h before addition of metronidazole and initiation of the experiment it is possible that even longer time is needed to reach steady state as apparent from Fig 1C. The steady state flux, J_ss_, displayed in Table 1 and Fig 1D was therefore calculated from the linear region between 6–24 h. The steady state flux illustrates a decrease in permeability for metronidazole through mucin gels with decreased water activity. No change or a very small decrease in the metronidazole flux is seen with decreased water activity for penetration through PEG gels and silicone membranes.

### Diffusion coefficient of fluorescein in mucin gels

In order to elucidate how water activities below 0.867 affect diffusion in mucin gels we investigated diffusion of the fluorophore fluorescein. We were able to control the hydration of the mucin films down to a water activity of 0.12 by incubation of the gels in humidity controlled environments. Mucin gels with a thickness of about 30–80 μm were obtained as shown in S1 Fig. In accordance with previously published results [16,23,27,28] micrometer sized structures were observed in the gels (S2 Fig).

**Fig 2:**
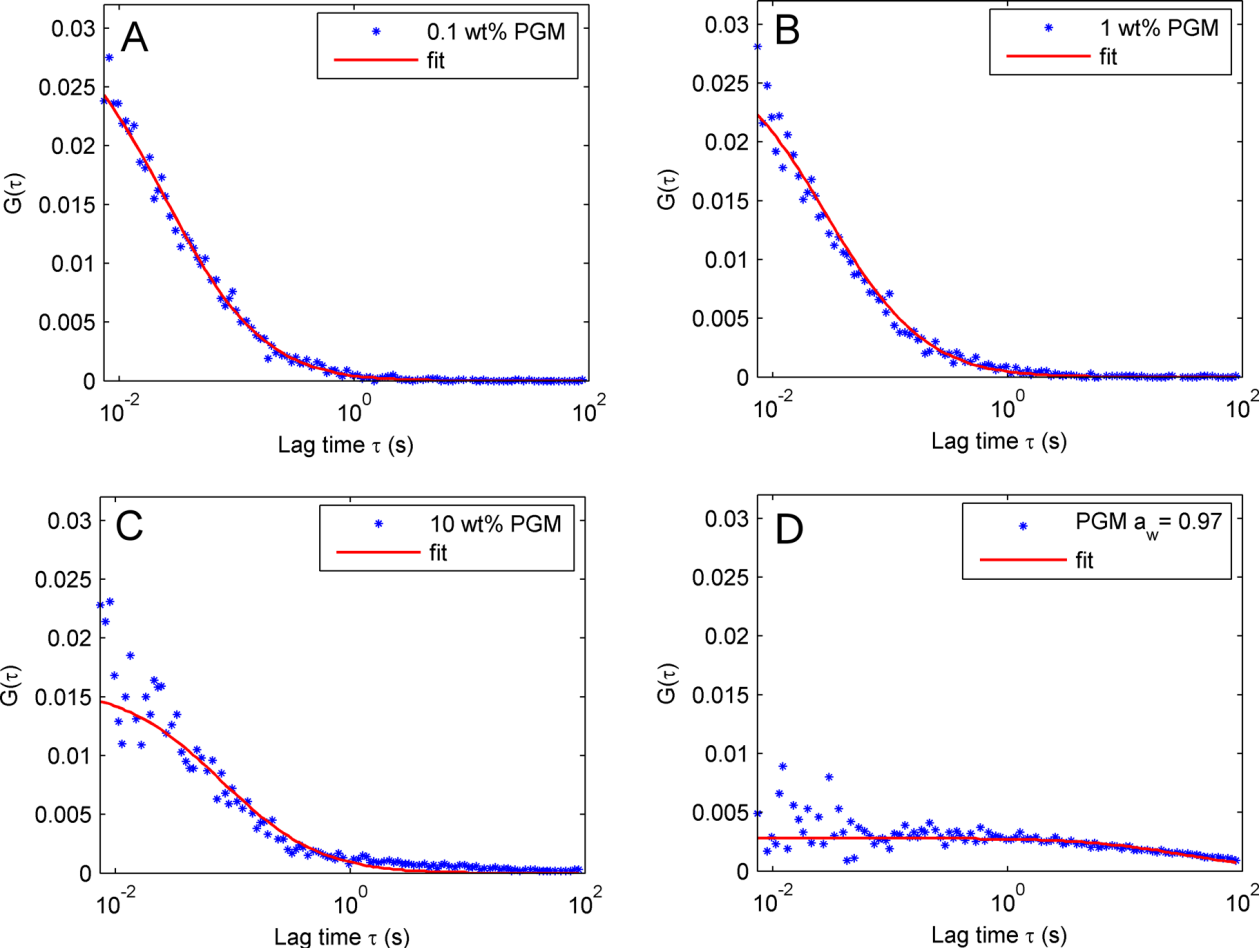
Representative FCS measurements on a mucin solution and a highly concentrated mucin film. Autocorrelation functions for measurements in (A) 0.1 wt% (B) 1.0 wt% (C) 10.0 wt% mucin solution and (D) concentrated mucin film with water activity of 0.97 are shown, together with fits with a model for pure diffusion.

Fluorescence correlation spectroscopy (FCS) measurements were used to measure diffusion coefficients of fluorescein in mucin gels and in dilute mucin solutions. Autocorrelation functions together with best fit are shown in Fig 2 for 0.1, 1.0, 10.0 wt% mucin solutions as well as for a mucin gel with a water activity of 0.97 containing approximately 40–60 wt% mucin. From the correlation curves it is clear that the diffusion coefficient drastically decreases with increased mucin concentration. It is clear that the autocorrelation function cannot accurately be fitted to a model considering only one diffusion process, as it contains several correlation times, see also S3 Fig. Nevertheless, the FSC measurements clearly demonstrate that the autocorrelation function, and thus diffusion characteristics of the florescence probe, largely differ between the different hydration conditions, Table 2. A large decrease in the diffusion coefficient with decreased water content is seen already in diluted mucin gels with a water activity close to one. The diffusion coefficient decreases further by several orders of magnitude as the water activity in the mucin gel decreases from 1 to 0.97.

**Table 2:** Approximate diffusion coeficients of flourescein in mucin gels obtained by FCS measurements.

**water activity**	**wt% mucin**	**D (μm**^**2**^**/s)**
1.0	0	510*
~1.0	0.1	550 ± 35
~1.0	1	459 ± 52
~1.0	10	121 ± 51
0.97	~50**	1.7 ± 1.5

* Value from literature [40,41] used to calibrate the confocal volume.

** Approximate mucin content obtained from Znamenskaya et al. [15]

**Fig 3:**
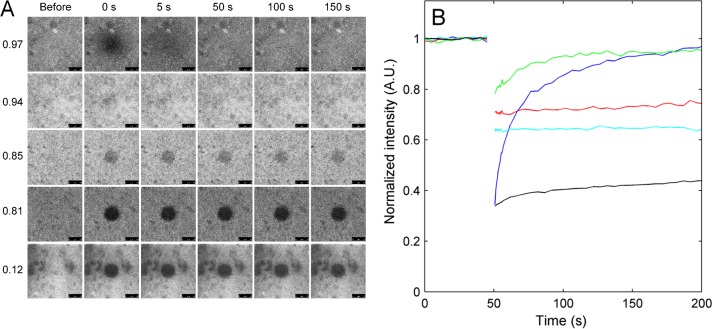
Fluorescence recovery after photobleaching of fluorescein in mucin gels. (A) Representative images before and after bleaching for mucin gels with water activities as indicated to the left and (B) representative normalized recovery curves obtained for gels with water activity of 0.97 (blue), 0.94 (green), 0.85 (red), 0.81 (cyan) and 0.12 (black) are shown. Scale bars in (A) are 10 μm.

Diffusion in concentrated mucin gels was further investigated using fluorescent recovery after photobleaching (FRAP), where the fluorescence recovery of a 10 μm spot was recorded after bleaching for 4 s. With this technique we were able to measure the diffusion in mucin gels with water activity between 0.97 and 0.12, to mucin gels with a concentration ranging from 50 wt% to 97 wt% (S1 Table). Representative images recorded before and after bleaching for selected FRAP measurements are shown in Fig 3A and representative recovery curves are shown in Fig 3B. For highly hydrated mucin gels, with water activities of 0.97 and 0.94, fast recovery was obtained while mucin gels with a lower water activity displayed a significantly lower recovery rate. Mucin gels with water activity of 0.85 and 0.81 display stable recovery curves with very little recovery (Fig 3B) while mucin gels with even lower water activity display a slight intensity increase in the recovery curves. However, due to experimental limitations we were not able to determine whether this intensity increase is due to diffusion of fluorophores or drift of samples during measurement.

**Fig 4:**
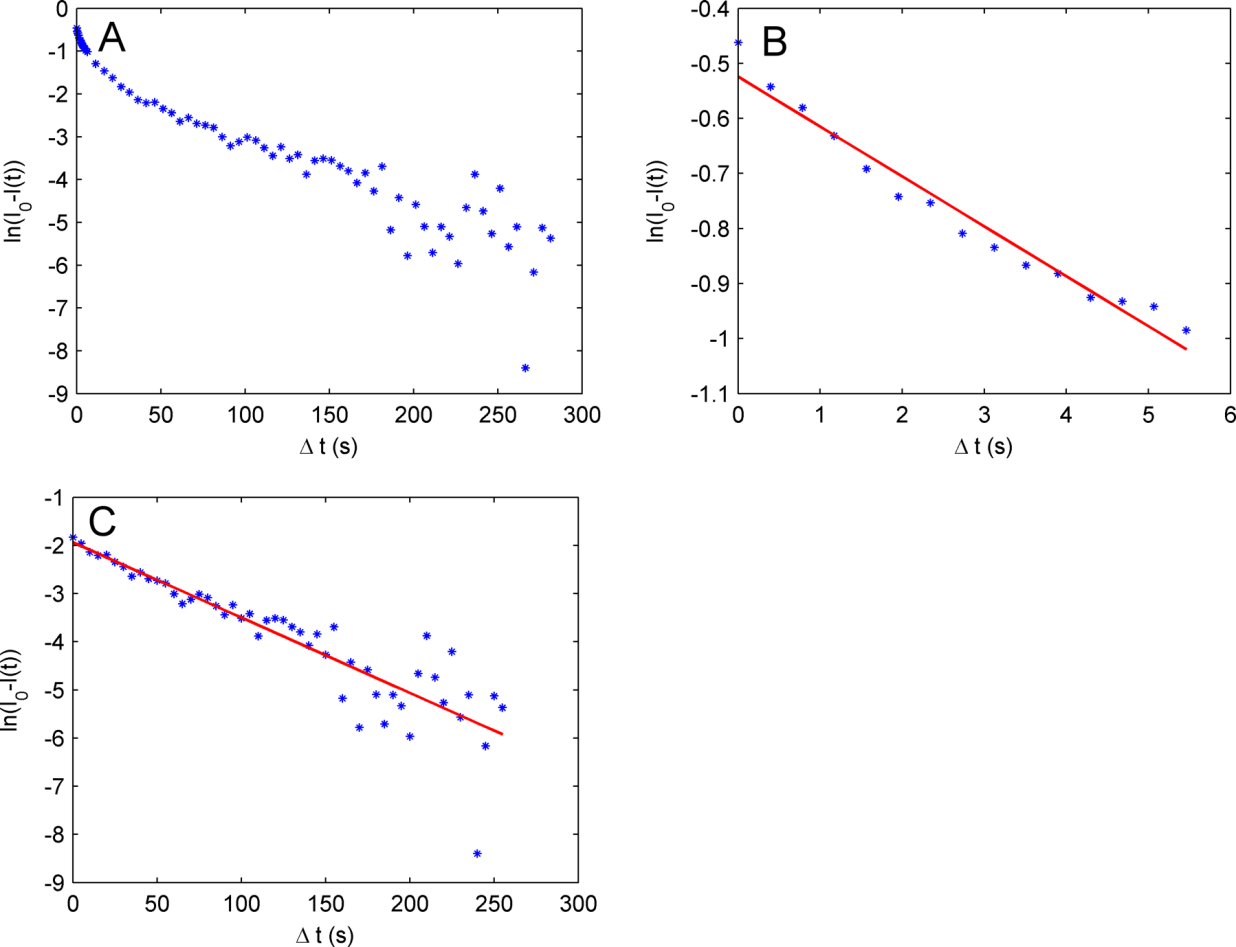
Data analysis of FRAP measurements. Representative normalized recovery curves performed by fitting the recovery curve to ln(I_0_-I(t)) = ln(I_0_-I_1_)-βt. (A) the complete measurement where two linear regimes are visible. The two linear regimes were used to calculate the diffusion coefficient of fluorescein for one fast (first 15 s after bleaching, (B) and one slow (from 15 s after bleaching, (C) diffusion process. The displayed data were obtained from mucin gels with a water activity of 0.97, but the same data treatment were performed on all the mucin gels studied (see S3–S5 Figs).

Recovery curves were modeled as a single exponential according to the function I(t)/I_0_ = 1-(I_0_-I_1_) e^-βt^ /I_0_. β was obtained as the slope of the linear regimes found when plotting ln(I_0_-I(t)) against t. Fig 4 displays representative plots of ln(I_0_-I(t)) against t for mucin gels with a water activity of 0.97 (A) and 0.12 (B). Representative curves for mucin gels with other water activities can be seen in S4–S6 Figs. Two linear regimes are present in most recovery curves where a fast process dominates the curve during the first 15 seconds after bleaching followed by a slower process 15 seconds after bleaching and onwards. This indicates that different dynamic processes contribute to the diffusion of fluorescein in mucin gels. Fits to the fast (0–15 s after bleaching, Fig 4C and 4D) and slow processes (15 s after bleaching to the end of the measurements, Fig 4E and 4F) were performed and the diffusion coefficients were calculated, S1 Table. Qualitatively a clear trend was observed with a dramatic reduction in diffusion coefficients with decreased water content in the mucin gels. For mucin gels with a water activity of 0.97 diffusion coefficients for fluorescein were determined to 0.638 ± 0.037 μm^2^/s and 0.141 ± 0.029 μm^2^/s while decreasing the water activity to 0.94 gave diffusion coefficients of 0.272 ± 0.034 μm^2^/s and 0.098 ± 0.038 μm^2^/s. For mucin gels with water activity 0.85 and lower the diffusion process is very slow and experimental limitations make it difficult to calculate the diffusion coefficients with sufficient accuracy. Nevertheless two diffusion processes were found with diffusion coefficients of < 0.1 μm^2^/s and <0.02 μm^2^/s.

## Discussion

From penetration studies as well as from both the FCS and FRAP experiments a decrease in the mobility of small molecules such as metronidazole and fluorescein is seen in mucin gels with decreased water activity, and thus decreased water content, in the mucin gels. In the FRAP experiments a drastic decrease in the diffusion coefficient of fluorescein in mucin gels can be seen when the water activity is lowered from 0.97 to 0.85. Similarly, the metronidazole flux though mucin films decreases when dehydrating from a water activity of 0.98 to 0.87.

### Metronidazole penetration through mucin gels and buccal mucosa

A decrease in the steady state flux of metronidazole through buccal mucosa with decreased water activity has previously been shown [33]. These results are in line with the current study where a decrease in steady state flux of metronidazole through mucin gels is seen with decreased water activity. Overall larger steady state fluxes are detected through buccal mucosa compared to the mucin films used in this study, which might be explained by the difference in barrier thicknesses between the experimental setups. The barrier thickness of buccal mucosa has been reported to be 282 to 294 μm [45,46], while a mucin film of about 1.74 mm was for practical reasons used in this study. Furthermore, mucus films exposed to air (e.g. nasal and buccal mucosa) are normally hydrated from the tissue below, creating a water gradient over the mucosal barrier. Due to the possibility for continuous hydration from inside of the body (in the case of in vitro experiments from the receptor compartment), it is possible that the buccal mucosa has a larger water activity than the environment it is exposed to. Indeed, studies have shown that while mucin gels become dry at 60–70% RH in vitro [15,16], in vivo the mucosa of the eyes do not become dry until subjected to 30% RH and the nasal cavities do not feel dry until exposed to 10% RH [30]. Also in the in vitro study of metronidazole diffusion through buccal mucosa [33] a water gradient over the barrier existed. Nevertheless, good agreement is obtained between metronidazole diffusion through mucin gel and buccal mucosa [33]. In both experiments the same trend is seen with a decrease in steady state flux with decreased water activity in the membranes.

It should be observed that we used commercially available pig gastric mucin from Sigma in this study, primarily because it allowed us to use the same sample batch in all experiments which would not have been possible using mucins from fresh scrapings due to the need for large sample volumes. We have also chosen to disperse the mucin into pure water as addition of salt would cause formation of salt crystals during the drying procedure. In previous study we have compared the water sorption of this commercial mucin with corresponding mucus freshly excised from pig, concluding that water sorption isotherms are relatively similar although the mucus absorbs less water in the commercial mucin at low relative humidities [16]. One reason for the lower water sorption of the mucus could be presence of impurities, such as enzymes, proteins and lipids. There is also some evidence in the literature that the viscosity of this commercially available mucin differs from that of excised pig gastric mucus [47]. Having said this, we believe that the advantages of using commercially available mucin as a model system for this study are larger than its limitations, none of the least when it comes to reproducibility of the results.

### Diffusion of fluorescein in mucin gels from FCS and FRAP measurements

For the autocorrelation functions obtained by FCS measurements on mucin solutions with 10 wt% mucin and the strongly concentrated mucin films, a non-zero convergence is observed. Furthermore, several of the autocorrelation functions display a second tail indicating that two diffusion processes with different rates are present. This is an indication that several diffusion processes take place simultaneously or that photobleaching of the fluorophores occur, as can be seen in the time traces for the concentrated mucin film in S7D Fig. Hence, diffusion constants cannot be described with one diffusion coefficient within the FCS measurements and the data were only used to obtain qualitative information on changes in the diffusion characteristics of the probe in the mucin gel at different conditions

In dilute mucin solutions the diffusion coefficient of fluorescein is of the same order of magnitude as diffusion of fluorescein in water. As the mucin concentration increases there is a large decrease in the diffusion constant, with largest decrease at high water content (0, 0.1, 1, 10 wt% mucin corresponding to aw close to unity). The results on dilute mucin solutions are in agreement with literature data where diffusion coefficients of 800–200 μm^2^/s were found in mucus and diluted mucin solutions (0–0.2 wt%) for molecules with sizes similar to sodium fluorescein [24,48].

For the concentrated mucin gel with a water activity of 0.97 the diffusion coefficient of fluorescein was evaluated with both FCS and FRAP. From FSC measurements a diffusion coefficient of 1.7 ± 1.5 μm^2^/s was obtained while two diffusion coefficients, 0.64 ± 0.04 μm^2^/s and 0.14 ± 0.03 μm^2^/s, were obtained in FRAP measurements. The two diffusion coefficients obtained in the FRAP experiments imply that several processes with different dynamics contribute to the diffusion of fluorescein in the mucin gels. Indeed for several of the mucin gels the autocorrelation function obtained by FCS contains more than one tail, which may indicate that several diffusion processes take place, see S3 Fig. As seen from the confocal microscopy images (Fig 3A and S2 Fig) the mucin gels are also largely heterogeneous with brighter and darker domains. In the dilute mucin solution (0.02–0.4 wt% mucin) the measured fluorescein self-diffusion coefficient (about 500 μm^2^/s) is similar to that of fluorescein in aqueous solution [40,41], and several orders of magnitude higher compared to the self-diffusion coefficient of mucin (2.5–0.5 μm^2^/s) [26]. Hence, fluorescein is not bound to the mucin proteins in diluted solutions, and we assume that fluorescein-mucin interaction does not change significantly with water content. This is also supported by the FCS measurements (S7 Fig), where we did not observe signal from large fluorescein labeled aggregate. It is likely that the slow diffusion process measured in FRAP measurements originates from the restricted diffusion of fluorescein molecules entrapped or caged in aqueous domains that are not connected and therefore do not allow long range diffusion. For mucin gels with water activity of 0.97 the fast diffusion process observed in the FRAP give a diffusion coefficient which is in the same range as the diffusion coefficient observed in FCS measurements taking into account the relatively large errors of the measurements. The FCS measurements register diffusion in a smaller physical volume and in shorter timescale compared to the FRAP measurements. This might explain that we only are able to measure the diffusion coefficient of the fast diffusion process of individual fluorescein molecules in most of the FCS measurements.

### Diffusion of metronidazole and fluorescein in mucin gels

**Fig 5:**
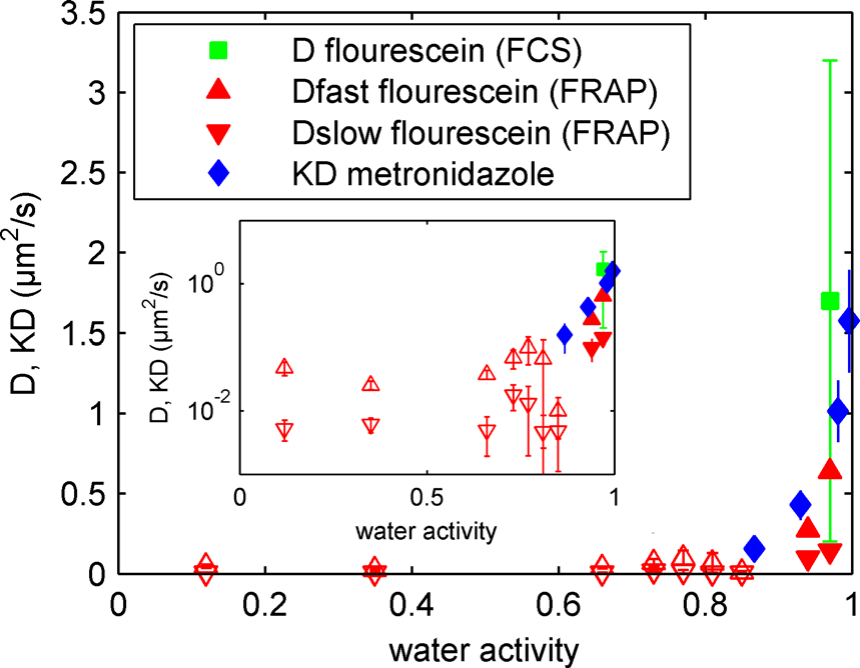
Diffusion in concentrated mucin gels. Diffusion of metronidazole through mucin gels shown in a lin-lin scaling and a log-lin scaling (insert). The diffusion was measured by penetration (blue diamonds) experiments and diffusion coefficient of fluorescein in mucin gels measured by FRAP (red triangles) and FCS (green square) experiments. The triangles and inverted triangles shows the fast and slow diffusion process, respectively, measured in FRAP experiment. Below water activity of 0.94, the diffusion of sodium fluorescein is not measurable under the current experimental conditions and the markers (open symbols) are only added to visualize the qualitative trend.

Both metronidazole and fluorescein are small molecules which are expected to diffuse through the mucin gels by same pathways. However, some differences between the molecules exist. Sodium fluorescein (M_w_ = 332 g/mol) is about twice as large as metronidazole (M_w_ = 171 g/mol) and structurally less flexible. In order to investigate how much these differences between the two molecules affect the transport properties through mucin gels we calculated the transport driven diffusion coefficients for metronidazole in mucin gels from the steady state flux according to Fick’s first law.

$$J=-D∙c_{i}^{m}\frac{\partial lna_{i}}{\partial z}=-D∙K∙c_{i}^{b}\frac{\partial lna_{i}}{\partial z}=-D∙K\frac{\Delta c_{i}^{b}}{\Delta z}\text{-}$$ (9)

where *a*_*i*_ is the activity of metronidazole, *K* is the partition coefficient of metronidazole between the donor and the gel/membrane, D is an effective diffusion coefficient of the drug/dye in the mucin layer, and *Δz* is the thickness of the gel/membrane. *c*_*i*_^*m*^ and *c*_*i*_^*b*^ is the concentration of metronidazole in the donor and the membrane, respectively. The last step in Eq (9) were obtained by assuming that the activity is linear with respect to the concentration (i. e. *a*_*i*_ *= c*_*i*_^*b*^*/c*_*i*_^*0*,*b*^ where *c*_*i*_^*0*,*b*^ is the solubility of metronidazole in the bulk of the donor solution, PEG solution). The thickness of the mucin gels were taken as 1.74 mm. As we do not know the partition coefficient of metronidazole between the donor solution and the mucin gels D*K is reported in Fig 5.

For both metronidazole and fluorescein a slower diffusion is seen with decreased water activity in the mucin gel. The effect is largest between water activities of 1 to 0.85, where a large decrease in the diffusion coefficient of fluorescein is seen as well as a large decrease in KD of metronidazole. For mucin gels with lower water activities (0.85 to 0.12) no large change in the diffusion coefficient of fluorescein is seen.

## Conclusion

Drug penetration experiments as well as FRAP and FCS measurements show that the mobility of small molecules such as metronidazole and fluorescein drastically decreases when the water activity in mucin gels decreases. Our results illustrate the possibility to alter the transport rate of molecules through mucus films by changing the water activity of the film, where only a small change is needed to obtain a large effect. This report shows the importance of considering how the water activity in the mucus films is changed when applying pharmaceutical formulations to the nose and eyes. For instance, addition of a mucoadhesive fomulation could dehydrate the mucosa [31] leading to a decreased diffusion in the mucin gel and hence reduce the drug uptake. This study thereby highlights the importance of considering how mucosal formulations affect the water content in the mucus film for optimizing the drug absorption.

## Supporting Information

S1 Fig Cross section of the mucin gels used for FRAP and FCS measurements. Mucin films obtained used in FRAP and FCS experiments displayed a thickness between 80–30 μm. B) Profile of the bleached area in fluorescence recovery after bleaching experiment. A cylinder with 10 μm diameter which covers most of the 30 μm thick film is bleached.
(PDF)

S2 Fig Confocal microscopy images of mucin gels. Image taken in A) fluorescence mode where fluorescence from fluorescein can be seen and B) reflection mode where mucin aggregates are visualized.
(PDF)

S3 Fig Representative FCS measurements on mucin solutions and highly concentrated mucin film. Aoutocorrelation function for measurements in A) 0.1 wt% B) 1.0 wt% C) 10.0 wt% mucin solution and D) concentrated mucin film with water activity of 0.97 are shown, together with fit with a model for pure diffusion.
(PDF)

S4 Fig Analysis of FRAP data. ln(I_0_-I(t)) vs Δt for representative recovery curves on mucin gels with water activity of 0.97 (A), 0.94 (B), 0.85 (C), 0.81 (D), 0.77 (E), 0.73 (F), 0.66 (G), 0.35 (H) and 0.12 (I). Two linear regimes are visible.
(PDF)

S5 Fig Fitting to the fast diffusion process observed in FRAP measurements. Analysis of fluorescence recovery rate for fluorescein dominating the recovery first 15 s after bleaching. Representative curves for mucin gels with water activity of 0.97 (A), 0.94 (B), 0.85 (C), 0.81 (D), 0.77 (E), 0.73 (F), 0.66 (G), 0.35 (H) and 0.12 (I) are shown.
(PDF)

S6 Fig Fitting to the slow diffusion process observed in FRAP measurements. Analysis of fluorescence recovery rate for fluorescein dominating the recovery first 15 s after bleaching. Representative curves for mucin gels with water activity of 0.97 (A), 0.94 (B), 0.85 (C), 0.81 (D), 0.77 (E), 0.73 (F), 0.66 (G), 0.35 (H) and 0.12 (I) are shown.
(PDF)

S7 Fig Time traces for the recorded intensity in the confocal volume in FCS measurements. Data A) 0.1 wt%, B) 1.0 wt%, C) 10.0 wt% mucin solutions and D) a mucin film equilibrated at 97% RH. No temporary large intensity fluctuations are seen indicating that no large scale fluorescent labeled aggregates temporary diffuse in or out of the confocal volume during the measurement, and hence we can assume that we are indeed measuring the diffusion of individual fluorophore molecules. For the strongly concentrated mucin gel (D), with slow diffusion of fluorescein molecules, an initial decrease in fluorescence intensity is seen showing that photo bleaching of the fluorescein molecules occur.
(PDF)

S1 Table Diffusion coeficients of sodium flourescein in mucin gels obtained by FRAP measuremetns. (PDF)
