# Supplementary material for: Diffusion through Pig Gastric Mucin: Effect of Relative Humidity
Source: PLoS One. 2016 Jun 23;11(6):e0157596. doi: 10.1371/journal.pone.0157596 (PMC4918968; doi:10.1371/journal.pone.0157596)
Supplement: S1 Fig — Mucin films obtained used in FRAP and FCS experiments displayed a thickness between 80–30 μm. B) Profile of the bleached area in fluorescence recovery after bleaching experiment. A cylinder with 10 μm diameter which covers most of the 30 μm thick film is bleached. (PDF) [file pone.0157596.s001.pdf]

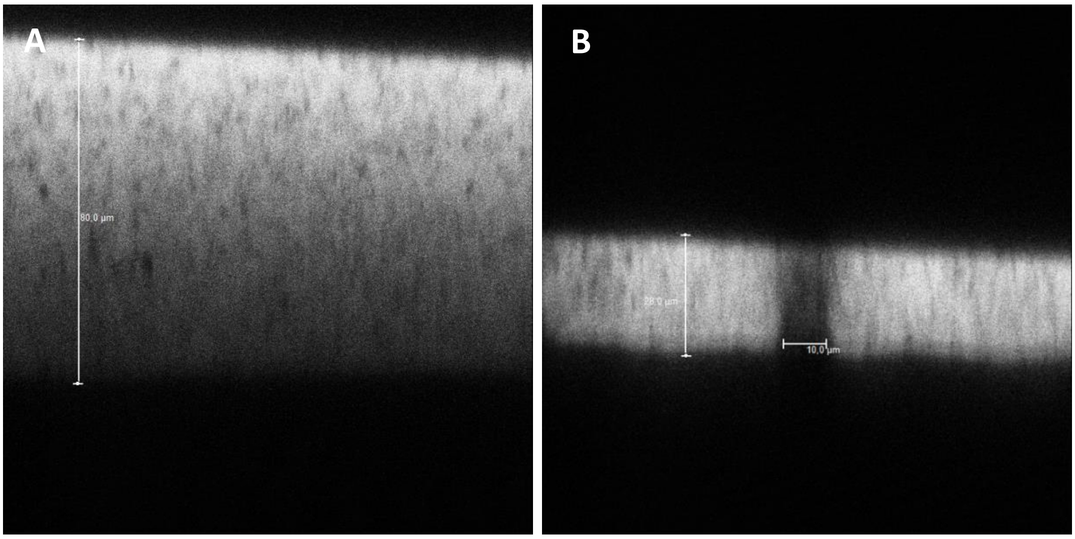

S1 Fig. Cross section of the mucin gels used for FRAP and FCS measurements. A) Mucin films obtained used in FRAP and FCS experiments displayed a thickness between 80-30 µm. B) Profile of the bleached area in fluorescence recovery after bleaching experiment. A cylinder with 10 µm diameter which covers most of the 30 µm thick film is bleached.
