# Supplementary material for: Diffusion through Pig Gastric Mucin: Effect of Relative Humidity
Source: PLoS One. 2016 Jun 23;11(6):e0157596. doi: 10.1371/journal.pone.0157596 (PMC4918968; doi:10.1371/journal.pone.0157596)
Supplement: S2 Fig — Image taken in A) fluorescence mode where fluorescence from fluorescein can be seen and B) reflection mode where mucin aggregates are visualized. (PDF) [file pone.0157596.s002.pdf]

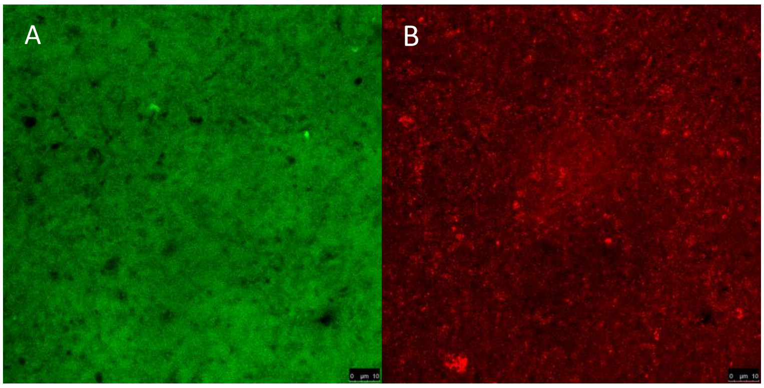

S2 Fig. Confocal microscopy images of mucin gels. Image taken in A) fluorescence mode where fluorescence from fluorescein can be seen and B) reflection mode where mucin aggregates are visualized.
