# Supplementary material for: Diffusion through Pig Gastric Mucin: Effect of Relative Humidity
Source: PLoS One. 2016 Jun 23;11(6):e0157596. doi: 10.1371/journal.pone.0157596 (PMC4918968; doi:10.1371/journal.pone.0157596)
Supplement: S3 Fig — Aoutocorrelation function for measurements in A) 0.1 wt% B) 1.0 wt% C) 10.0 wt% mucin solution and D) concentrated mucin film with water activity of 0.97 are shown, together with fit with a model for pure diffusion. (PDF) [file pone.0157596.s003.pdf]

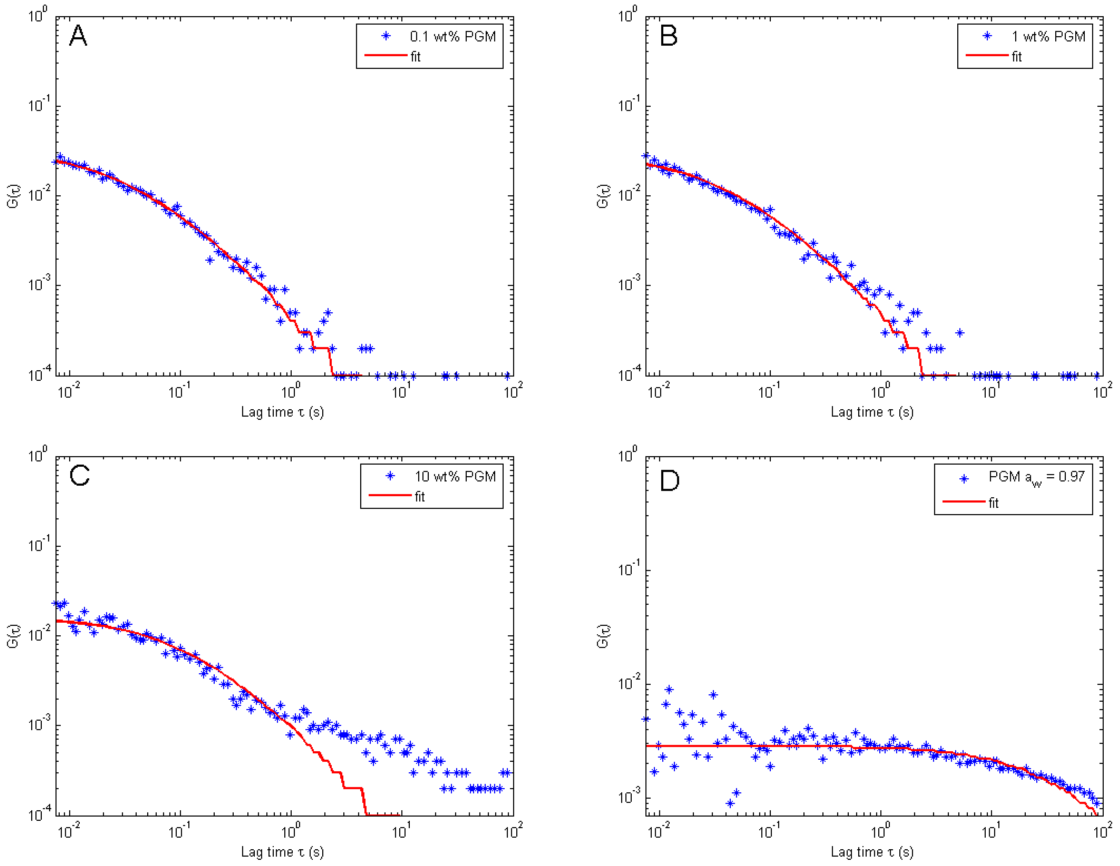

S3 Fig. Representative FCS measurements on mucin solutions and highly concentrated mucin film. Aoutocorrelation function for measurements in A) 0.1 wt% B) 1.0 wt% C) 10.0 wt% mucin solution and D) concentrated mucin film with water activity of 0.97 are shown, together with fit with a model for pure diffusion.
