# Supplementary material for: Diffusion through Pig Gastric Mucin: Effect of Relative Humidity
Source: PLoS One. 2016 Jun 23;11(6):e0157596. doi: 10.1371/journal.pone.0157596 (PMC4918968; doi:10.1371/journal.pone.0157596)
Supplement: S4 Fig — ln(I0-I(t)) vs Δt for representative recovery curves on mucin gels with water activity of 0.97 (A), 0.94 (B), 0.85 (C), 0.81 (D), 0.77 (E), 0.73 (F), 0.66 (G), 0.35 (H) and 0.12 (I). Two linear regimes are visible. (PDF) [file pone.0157596.s004.pdf]

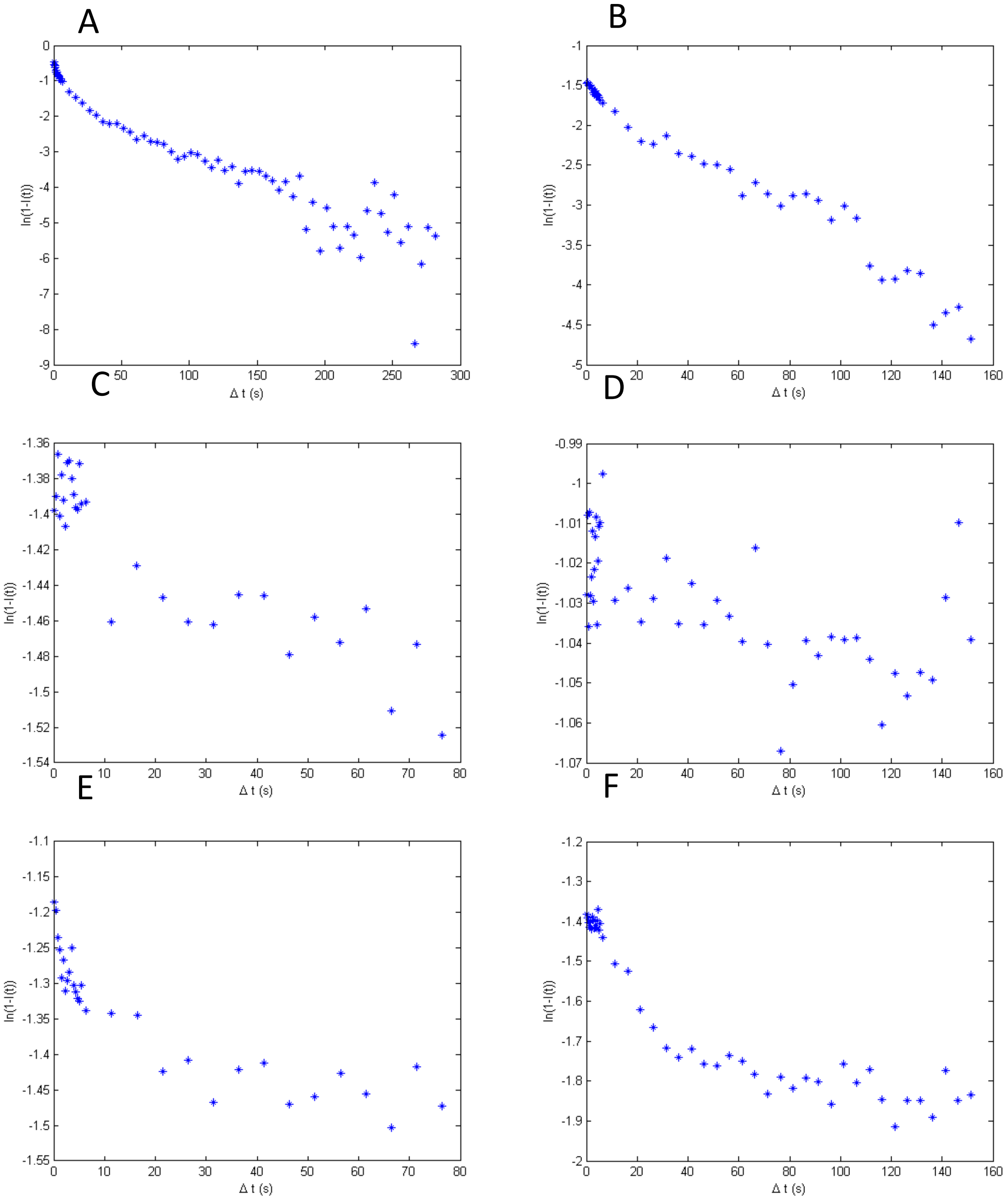
A
B
C
D
E
F
ln(1-I(t))
Δ t (s)

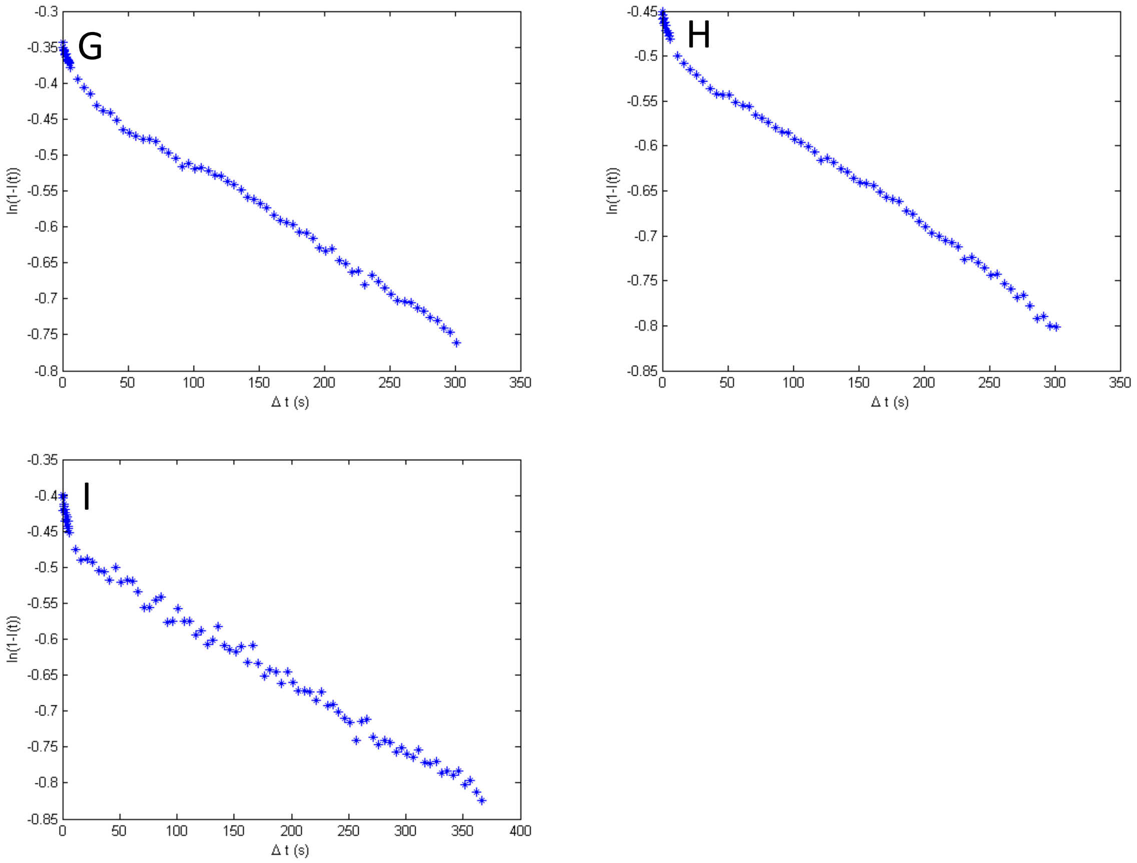

S4 Fig. Analysis of FRAP data. $\ln(I_0 - I(t))$ vs Δt for representative recovery curves on mucin gels with water activity of 0.97 (A), 0.94 (B), 0.85 (C), 0.81 (D), 0.77 (E), 0.73 (F), 0.66 (G), 0.35 (H) and 0.12 (I). Two linear regimes are visible.
