# Supplementary material for: Diffusion through Pig Gastric Mucin: Effect of Relative Humidity
Source: PLoS One. 2016 Jun 23;11(6):e0157596. doi: 10.1371/journal.pone.0157596 (PMC4918968; doi:10.1371/journal.pone.0157596)
Supplement: S5 Fig — Analysis of fluorescence recovery rate for fluorescein dominating the recovery first 15 s after bleaching. Representative curves for mucin gels with water activity of 0.97 (A), 0.94 (B), 0.85 (C), 0.81 (D), 0.77 (E), 0.73 (F), 0.66 (G), 0.35 (H) and 0.12 (I) are shown. (PDF) [file pone.0157596.s005.pdf]

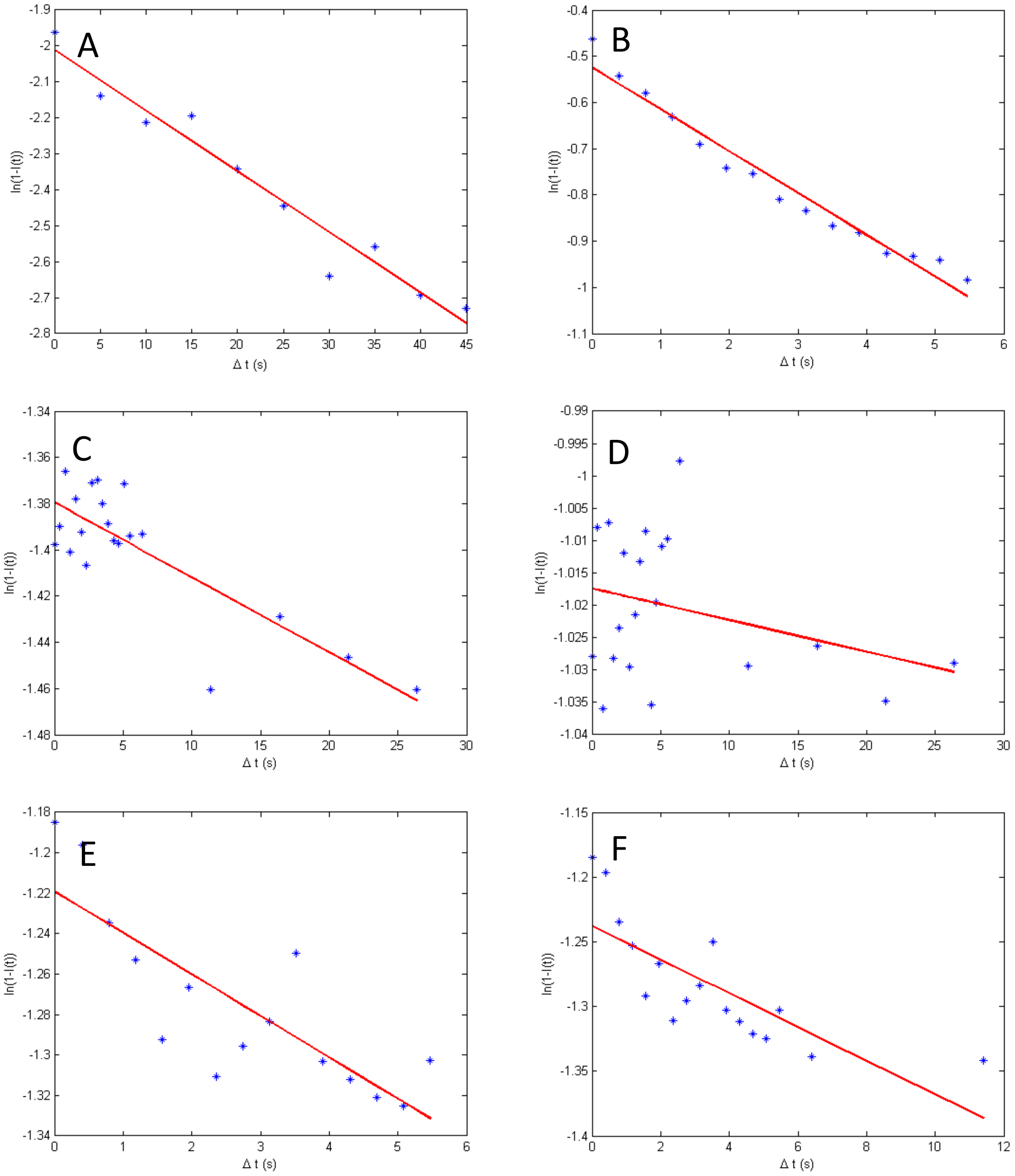
A
B
C
D
E
F
ln(1-I(t))
Δ t (s)

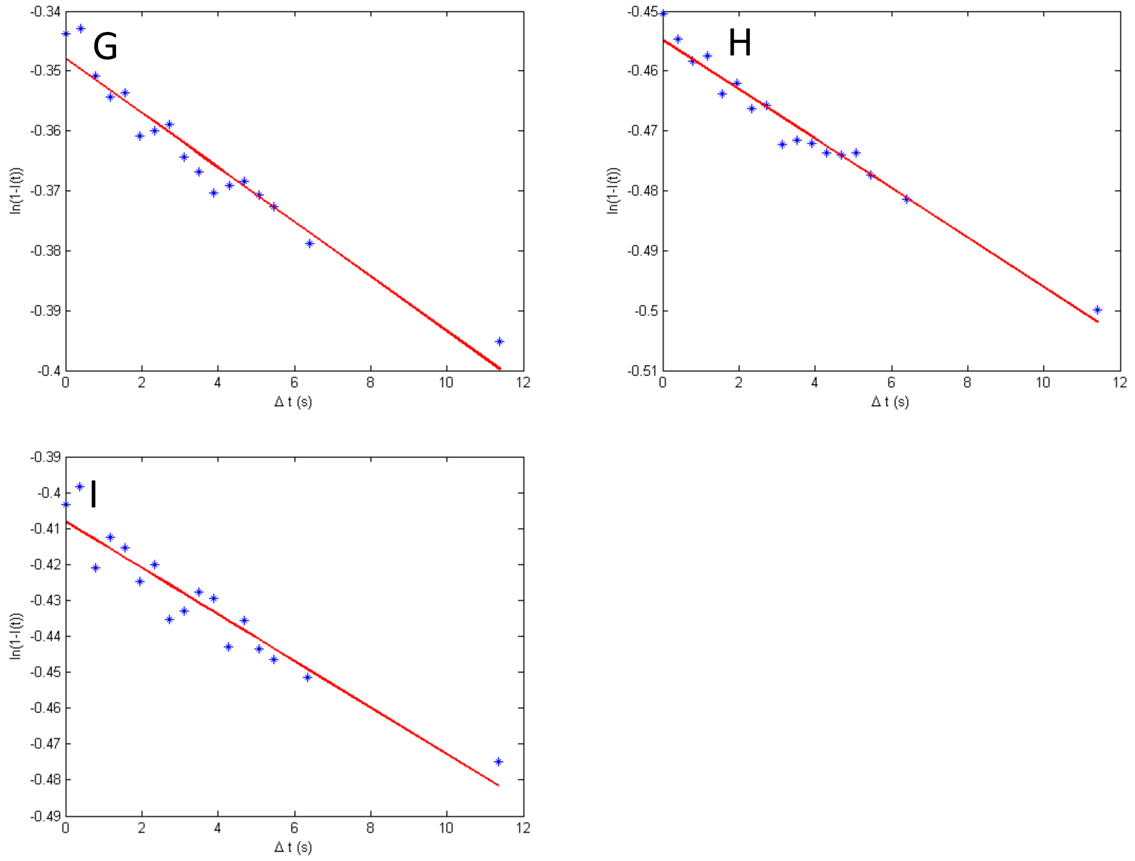

S5 Fig. Fitting to the fast diffusion process observed in FRAP measurements. Analysis of fluorescence recovery rate for fluorescein dominating the recovery first 15 s after bleaching. Representative curves for mucin gels with water activity of 0.97 (A), 0.94 (B), 0.85 (C), 0.81 (D), 0.77 (E), 0.73 (F), 0.66 (G), 0.35 (H) and 0.12 (I) are shown.

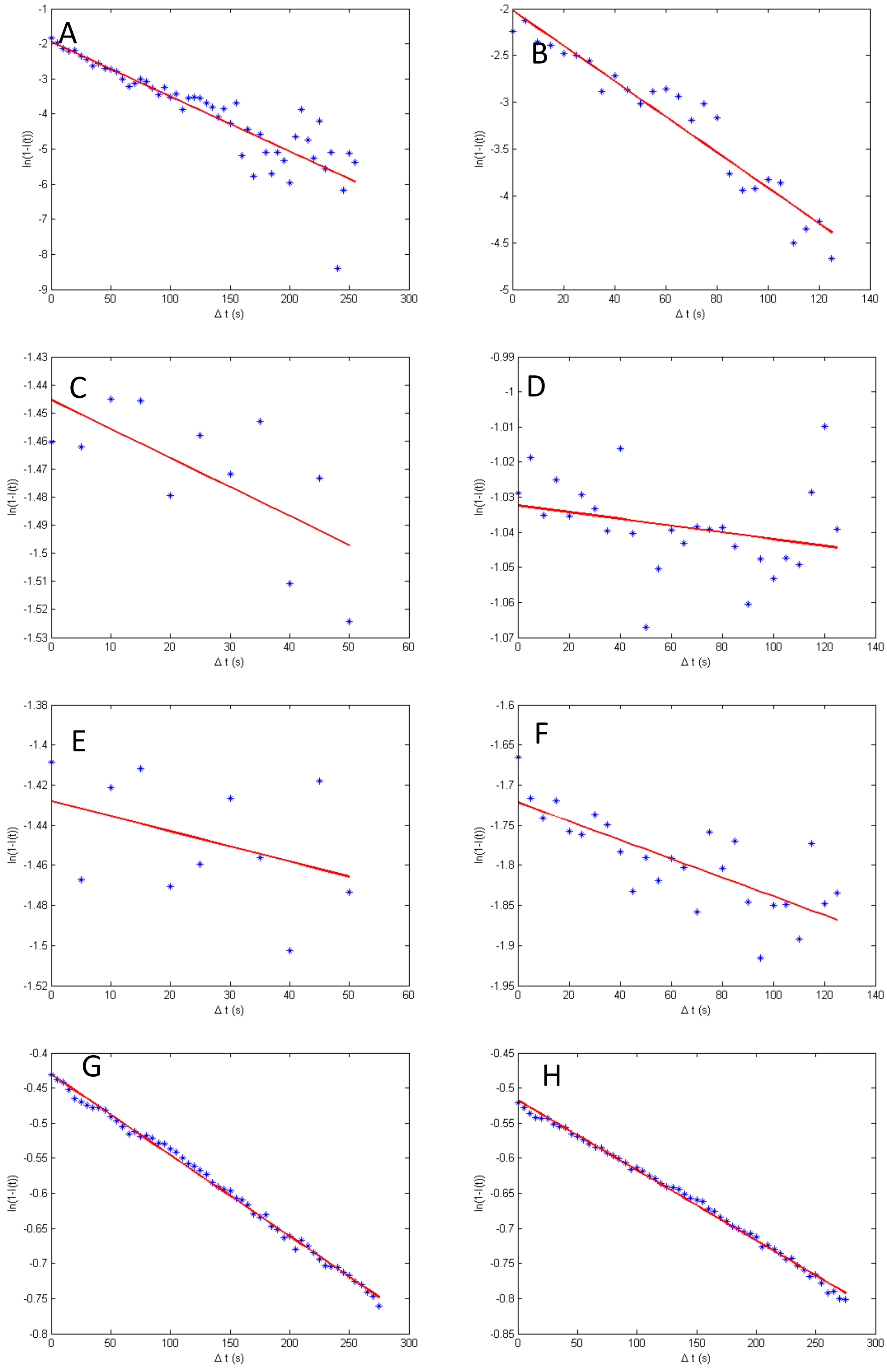
A
B
C
D
E
F
G
H
ln(1-I(t))
Δ t (s)

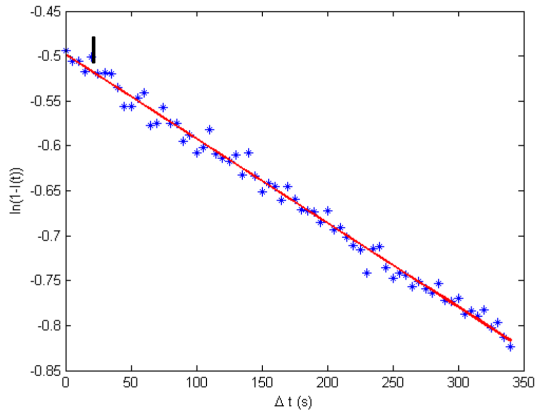

S6 Fig. Fitting to the slow diffusion process observed in FRAP measurements. Analysis of fluorescence recovery rate for fluorescein dominating the recovery from 15 s after bleaching. Representative curves for mucin gels with water activity of 0.97 (A), 0.94 (B), 0.85 (C), 0.81 (D), 0.77 (E), 0.73 (F), 0.66 (G), 0.35 (H) and 0.12 (I) are shown.

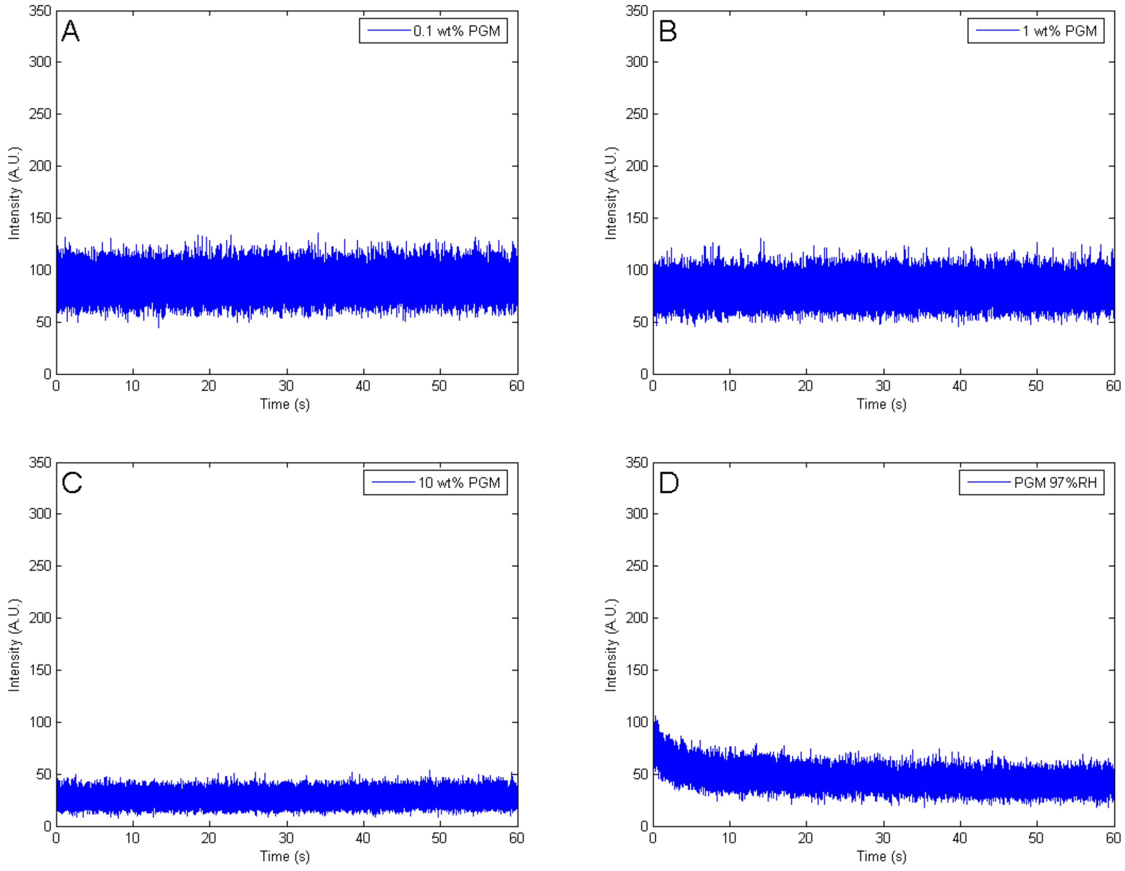

S7 Fig. Time traces for the recorded intensity in the confocal volume in FCS measurements. Data A) 0.1 wt%, B) 1.0 wt%, C) 10.0 wt% mucin solutions and D) a mucin film equilibrated at 97% RH. No temporary large intensity fluctuations are seen indicating that no large scale fluorescent labeled aggregates temporary diffuse in or out of the confocal volume during the measurement, and hence we can assume that we are indeed measuring the diffusion of individual fluorophore molecules. For

the strongly concentrated mucin gel (D), with slow diffusion of fluorescein molecules, an initial decrease in fluorescence intensity is seen showing that photo bleaching of the fluorescein molecules occur.

S1 Table. Diffusion coeficients of sodium flourescein in mucin gels obtained by FRAP measuremetns.

water activity	wt% mucin *	Osmotic pressure (MPa)	D_{fast} (µm^2/s)	D_{slow} (µm^2/s)
0.97	~50*	4.3	0.638 ± 0.037	0.141 ± 0.029
0.94	63	8.7	0.272 ± 0.034	0.098 ± 0.038
0.85	68	22.7	< 0.1	< 0.02
0.81	77	29.5	< 0.1	< 0.02
0.77	80	36.6	< 0.1	< 0.02
0.73	82	44.0	< 0.1	< 0.02
0.66	85	58.1	< 0.1	< 0.02
0.35	91	146.8	< 0.1	< 0.02
0.12	95	296.6	< 0.1	< 0.02

*Mucin concentration is calculated from water sorption isotherms previously reported (Znamenskaya et al. *J Phys Chem B* 2012 116(16) 5047-5055 and Znamenskaya et al. *J Phys Chem B* 2013 117(8):2554-2563).
