# Supplementary material for: Diffusion through Pig Gastric Mucin: Effect of Relative Humidity
Source: PLoS One. 2016 Jun 23;11(6):e0157596. doi: 10.1371/journal.pone.0157596 (PMC4918968; doi:10.1371/journal.pone.0157596)
Supplement: S6 Fig — Analysis of fluorescence recovery rate for fluorescein dominating the recovery first 15 s after bleaching. Representative curves for mucin gels with water activity of 0.97 (A), 0.94 (B), 0.85 (C), 0.81 (D), 0.77 (E), 0.73 (F), 0.66 (G), 0.35 (H) and 0.12 (I) are shown. (PDF) [file pone.0157596.s006.pdf]

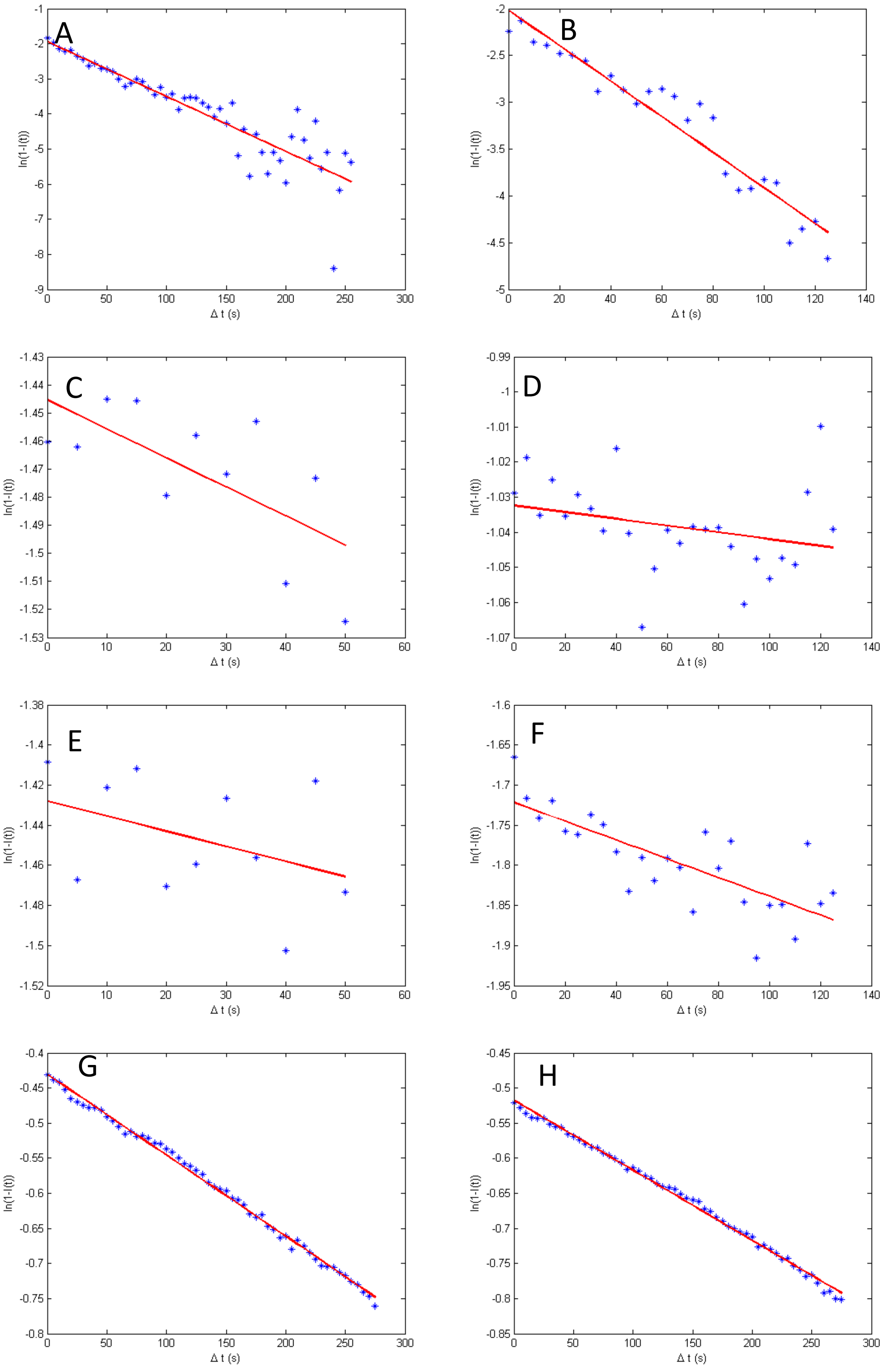
A
B
C
D
E
F
G
H
ln(1-I(t))
Δ t (s)

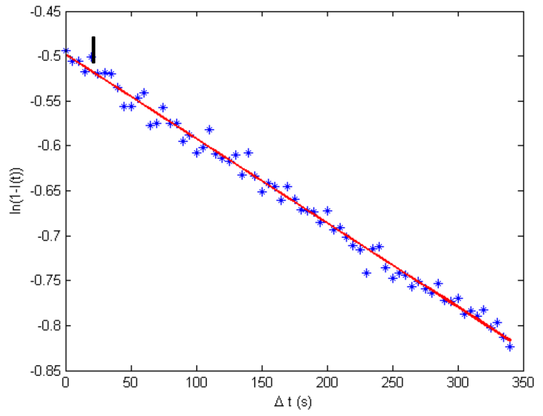

S6 Fig. Fitting to the slow diffusion process observed in FRAP measurements. Analysis of fluorescence recovery rate for fluorescein dominating the recovery from 15 s after bleaching. Representative curves for mucin gels with water activity of 0.97 (A), 0.94 (B), 0.85 (C), 0.81 (D), 0.77 (E), 0.73 (F), 0.66 (G), 0.35 (H) and 0.12 (I) are shown.
