# Supplementary material for: Diffusion through Pig Gastric Mucin: Effect of Relative Humidity
Source: PLoS One. 2016 Jun 23;11(6):e0157596. doi: 10.1371/journal.pone.0157596 (PMC4918968; doi:10.1371/journal.pone.0157596)
Supplement: S7 Fig — Data A) 0.1 wt%, B) 1.0 wt%, C) 10.0 wt% mucin solutions and D) a mucin film equilibrated at 97% RH. No temporary large intensity fluctuations are seen indicating that no large scale fluorescent labeled aggregates temporary diffuse in or out of the confocal volume during the measurement, and hence we can assume that we are indeed measuring the diffusion of individual fluorophore molecules. For the strongly concentrated mucin gel (D), with slow diffusion of fluorescein molecules, an initial decrease in fluorescence intensity is seen showing that photo bleaching of the fluorescein molecules occur. (PDF) [file pone.0157596.s007.pdf]

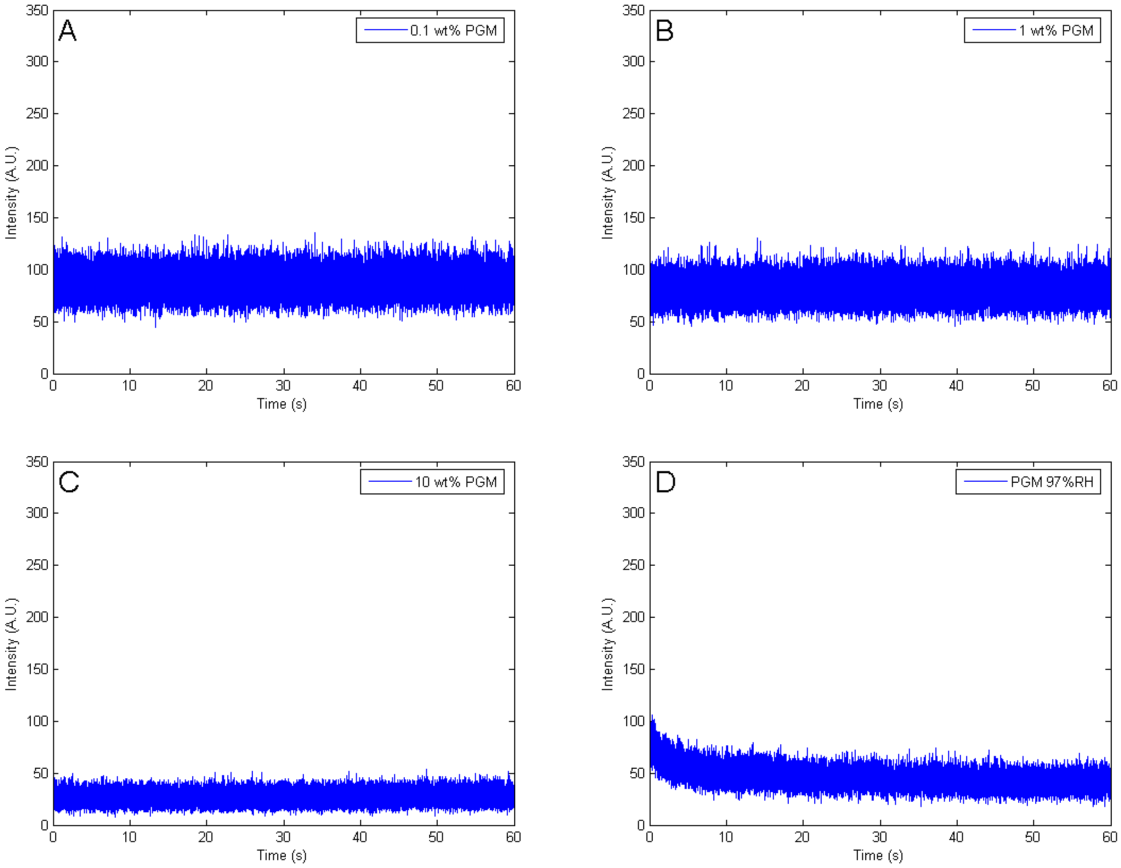

S7 Fig. Time traces for the recorded intensity in the confocal volume in FCS measurements. Data A) 0.1 wt%, B) 1.0 wt%, C) 10.0 wt% mucin solutions and D) a mucin film equilibrated at 97% RH. No temporary large intensity fluctuations are seen indicating that no large scale fluorescent labeled aggregates temporary diffuse in or out of the confocal volume during the measurement, and hence we can assume that we are indeed measuring the diffusion of individual fluorophore molecules. For the strongly concentrated mucin gel (D), with slow diffusion of fluorescein molecules, an initial decrease in fluorescence intensity is seen showing that photo bleaching of the fluorescein molecules occur.
