# Supplementary material for: Diffusion through Pig Gastric Mucin: Effect of Relative Humidity
Source: PLoS One. 2016 Jun 23;11(6):e0157596. doi: 10.1371/journal.pone.0157596 (PMC4918968; doi:10.1371/journal.pone.0157596)
Supplement: S1 Table — (PDF) [file pone.0157596.s008.pdf]

S1 Table. Diffusion coeficients of sodium flourescein in mucin gels obtained by FRAP measuremetns.

water activity	wt% mucin *	Osmotic pressure (MPa)	D_{fast} (µm^2/s)	D_{slow} (µm^2/s)
0.97	~50*	4.3	0.638 ± 0.037	0.141 ± 0.029
0.94	63	8.7	0.272 ± 0.034	0.098 ± 0.038
0.85	68	22.7	< 0.1	< 0.02
0.81	77	29.5	< 0.1	< 0.02
0.77	80	36.6	< 0.1	< 0.02
0.73	82	44.0	< 0.1	< 0.02
0.66	85	58.1	< 0.1	< 0.02
0.35	91	146.8	< 0.1	< 0.02
0.12	95	296.6	< 0.1	< 0.02

*Mucin concentration is calculated from water sorption isotherms previously reported (Znamenskaya et al. *J Phys Chem B* 2012 116(16) 5047-5055 and Znamenskaya et al. *J Phys Chem B* 2013 117(8):2554-2563).
